# Effect of Adherence to the Mediterranean Diet on Maternal Iron Related Biochemical Parameters during Pregnancy and Gestational Weight Gain

**DOI:** 10.3390/life13051138

**Published:** 2023-05-08

**Authors:** María Morales-Suárez-Varela, Isabel Peraita-Costa, Alfredo Perales-Marín, Beatriz Marcos Puig, Juan Llopis-Morales, Yolanda Picó

**Affiliations:** 1 Research Group in Social and Nutritional Epidemiology, Pharmacoepidemiology and Public Health, Department of Preventive Medicine and Public Health, Food Sciences, Toxicology and Forensic Medicine, Faculty of Pharmacy, Universitat de València, Av. Vicent Andrés Estelles s/n, 46100 Burjassot, València, Spain; 2 Biomedical Research Center in Epidemiology and Public Health Network, Carlos III Health Institute, Av. Monforte de Lemos 3-5 Pabellón 11 Planta 0, 28029 Madrid, Madrid, Spain; 3 Department of Gynecology and Obstetrics, La Fé University and Polytechnic Hospital, Avda. Fernando Abril Martorell, 106, 46026 València, Valencia, Spain; 4 Environmental and Food Safety Research Group (SAMA-UV), Desertification Research Centre, (CIDE, CSIC-UV-GV), Moncada-Naquera Road Km 4.5, 46113 Moncada, Valencia, Spain

**Keywords:** pregnancy, Mediterranean diet, anemia, iron

## Abstract

Gestation is a crucial life stage for both women and offspring, and outcomes are affected by many environmental factors, including diet. The Mediterranean dietary pattern (MD) is considered a healthy eating pattern that can provide the nutritional requirements of pregnancy. Meanwhile, iron deficiency anemia is one of the most frequent complications related to pregnancy. This study aimed to evaluate how the level of adherence to the MD influences maternal gestational weight gain and specific iron-related maternal biochemical parameters during the pregnancy. Accordingly, an observational, population-based study using data from pregnant women conducted over the entire course of their pregnancy was carried out. Adherence to the MD was assessed once using the MEDAS score questionnaire. Of the 506 women studied, 116 (22.9%) were classified as demonstrating a high adherence, 277 (54.7%) a medium adherence, and 113 (22.3%) a low adherence to the MD. No differences were observed in gestational weight gain among the MD adherence groups but the adequacy of weight gain did vary among the groups, with the proportions of inadequate (insufficient or excessive) weight gain presenting the most notable differences. Total anemia prevalence was 5.3%, 15.6%, and 12.3%, respectively, during the first, second, and third trimesters. For iron-related biochemical parameters, no differences are observed among the adherence groups during pregnancy. With high adherence to the MD as the reference group, the crude odds of iron deficiency diagnosis are significant in the first trimester for both the medium [OR = 2.99 (1.55–5.75)] and low [OR = 4.39 (2.15–8.96)] adherence groups, with deficient adherence to the Mediterranean dietary pattern being responsible for 66.5% (35.5–82.6) and 77.2% (53.5–88.8) of the risk of iron deficiency diagnosis for medium and low adherence, respectively. However, adjusted odds ratios were not significant, possibly due to the small sample size. Our data suggest that MD adherence could be related to gestational weight gain adequacy and that optimal adherence could reduce iron deficiency and/or anemia during pregnancy in the studied population.

## 1. Introduction

Pregnancy is a very particular stage during a woman’s life that can be affected by many healthcare and lifestyle factors. Maintaining healthy habits such as eating well is always important, but especially during pregnancy as it helps support maternal wellbeing and supplies the nutrition needed for correct fetal growth and development. Nutritional deficiencies can be exacerbated during pregnancy due to increased nutritional demands [1]. It has been demonstrated that an inability to fulfil these increased demands is associated with potentially adverse effects on the mother and the fetus, such as fetal growth abnormalities, cardiovascular disease, hypertension, type 2 diabetes, and/or anemia [2,3,4]. Maternal malnutrition may also have intergenerational effects as it has been shown to affect not only short-term but also more long-term health outcomes in children [5].

The study of dietary patterns has emerged as an alternative to the study of single nutrient intakes to examine the relationship between diet and health. Several advantages have been pointed out, some of them conceptual, since combinations of foods could reflect food preferences modulated by sociocultural or environmental determinants, and other methodological ones, such as detecting cumulative effects of different nutrients included in the same pattern. The Mediterranean dietary pattern (MD) has been recognized as one of the healthiest eating patterns and is characterized by its high content of fruits, vegetables, nuts, legumes, whole grains, and fish, as well as its low percentages of red meat, meat derivatives, refined cereals, and sugars. Even though the health benefits of the MD have been demonstrated in previous experimental studies [6,7,8,9], the underlying mechanisms have not been elucidated, and individual metabolic responses may differ.

The recommended gestational weight gain amounts for singleton pregnancies given in the 2009 Institute of Medicine (IOM) guidelines are 12.5–18 kg, 11.5–16 kg, 7–11.5 kg, and 5–9 kg for women with pre-pregnancy BMIs classified as underweight, normal weight, overweight, and obese, respectively [10]. The global prevalence of gestational weight gain above and below these guidelines has been estimated at 27.8% (95% CI; 26.5, 29.1) and 39.4% (95% CI; 37.1, 41.7), respectively [11]. Several maternal and infant health problems have been related to inadequate gestational weight gain, such as gestational diabetes and preeclampsia, instrumental or cesarean delivery, inadequate weight in gestational-age infants, or lower cognitive skills [11]. The high prevalence of inadequate gestational weight gain may be a consequence of several factors such as lifestyle changes during pregnancy, psychological and social maternal influences, and the recent global nutritional transition [11].

Iron deficiency is the most common nutritional deficiency worldwide [12,13], and women are especially at risk, particularly during pregnancy [14]. The symptoms and clinical signs of iron deficiency are usually of moderate intensity and often neglected, especially since they are not very specific and can be confused with those of other diseases [15]. This is particularly true of the main symptom, fatigue [16]. However, iron deficiency has also been associated with more severe consequences, such as impaired physical function [17,18] and reduced quality of life [19,20]. Over time, iron deficiency will lead to iron deficiency anemia if corrective measures are not taken. Anemia is a condition in which you lack enough healthy red blood cells to carry adequate oxygen to your body’s tissues. There are many forms of anemia, each with its own cause, but iron deficiency anemia is the most common type [21] and that to which this study refers. In Spain, 13.4% of women of reproductive age (15–49 years old) and one fifth of pregnant women have anemia (<12 g/dL for non-pregnant women and <11 g/dL for pregnant women) [22]. The presence of iron deficiency anemia during pregnancy is associated with adverse maternal–fetal health outcomes, such as preterm delivery, low birthweight, and decreased iron stores.

This study aimed to evaluate how the level of adherence to the MD influences maternal gestational weight gain and specific iron-related maternal biochemical parameters during pregnancy.

## 2. Materials and Methods

This research was performed in accordance with the ethical standards outlined in the 1964 Declaration of Helsinki and its later amendments. Institutional review board approval was obtained from the Clinical Research Ethical Committee of the La Fe University and Polytechnic Hospital (Ethic Committee N 2014/0116 of 8 September 2014). Data collection was performed according to Organic Law 3/2018 of December 5th regarding the Protection of Data of an Official Nature in order to guarantee confidentiality.

### 2.1. Study Design

This was an observational, population-based, periodic, cross-sectional study that used information from women who were patients of the Valencia-La Fe Health Department (Valencia, Spain).

The department includes 20 primary care centers, a health center, a specialty center, and a hospital, La Fe University and Polytechnic Hospital. This public National Health Service hospital is responsible for the care of the 300,000 inhabitants of the department, and in the years 2020 and 2021, attended to 4307 and 4126 births, respectively.

### 2.2. Study Population

An a priori power analysis was performed to determine the sample size required to test the study hypothesis: that the level of adherence to the MD during pregnancy may affect the prevalence of iron-related conditions such as anemia, which in European pregnant women has been estimated to be between 21–35% [23]. The sample size calculation was made using the Ene 3.0 program with power of 0.97, and an alpha level of 0.05. An estimated prevalence of iron deficiency and/or anemia of 40% yielded a required sample size of 203 women.

The following inclusion criteria were established: women must have given birth at the La Fe University and Polytechnic Hospital in 2020 or 2021 and have been treated from pregnancy confirmation before 12 weeks of gestation through to the six-week post-partum visit at the department, with at least one planned and recorded visit per trimester; birth must have been of a live singleton; electronic medical records from at least 4 different visits during the antenatal (one per trimester) and perinatal (at or within 5 days post birth) periods must have been available; and personal characteristics, habits, and dietary intake data must have been available. The exclusion criteria were: multiple pregnancy; fetal loss at any time during pregnancy up to and including birth. If during the 2-year span of the study a woman was eligible for inclusion on more than one occasion, only one pregnancy would be considered.

**Figure 1:**
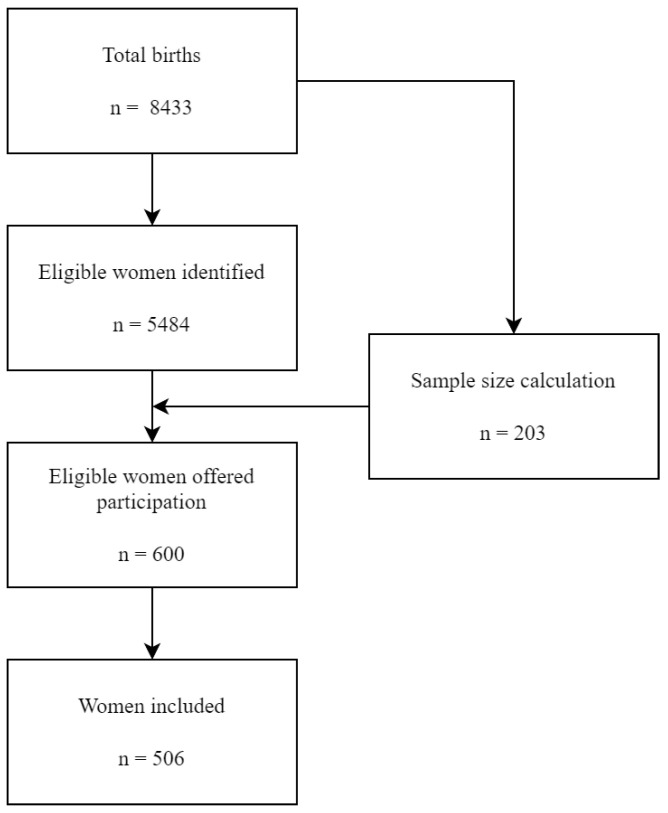
Recruitment and selection process.

As the previous sample size calculation indicated that a minimum of ~200 participants would be necessary, six hundred eligible women were identified through a simple random sampling method of the women in the Maternity Ward of the La Fe University and Polytechnic Hospital and invited to participate in this study during a post-birth visit, considering the potential participation rate. After excluding those who did not consent or gave incomplete responses, 506 women were included in the final analysis, equivalent to a 84.3% response rate (Figure 1). Written informed consent was obtained from all individual participants included in the study.

### 2.3. Data Collection

Data collection was conducted in two phases. First, during the last programed antenatal visit (36–38 weeks), data on personal characteristics and lifestyle were collected. This was achieved through a face-to-face interview with a trained interviewer. Subsequently, electronic medical records were reviewed, and relevant anthropometric and medical data were retrieved.

#### 2.3.1. Personal and Medical Data

The personal data collected include but were not limited to: age; origin; gestational age; obstetric history; drug, alcohol, and tobacco exposure; biochemical parameters; and supplement intake.

Regarding anthropometric data, height was recorded, weight pre-pregnancy and in the 1st, 2nd and 3rd trimesters was retrieved, and BMI was calculated for pre-pregnancy and in the 1st, 2nd and 3rd trimesters. Total gestational weight gain was also calculated, and adequacy was determined following the Institute of Medicine Guidelines [10].

Data on maternal physical activity during pregnancy were collected using a questionnaire where women self-reported their habitual activity throughout pregnancy. The questionnaire was modeled on the physical activity section of the Spanish National Health Survey, which is based on the International Physical Activity Questionnaire (IPAQ) [24]. Women were classified into the four levels of physical activity: None (completely sedentary), Low, Moderate, and High.

The threshold values for a diagnosis of iron deficiency and/or anemia were: Hb level < 11 g/dL in the 1st and 3rd trimesters; Hb level < 10.5 g/dL in the 2nd trimester; Ferritin level < 30 g/L [25].

#### 2.3.2. Adherence to the Mediterranean Dietary Pattern

The Mediterranean Diet Adherence Screener (MEDAS) was used to assess adherence to the MD. The MEDAS questionnaire was created as part of the PREDIMED project, and its development and validation have been detailed in previous publications [26]. The questionnaire includes 14 items that allow a quick and simple assessment of dietary habits associated with the MD in Spain. Each item is scored individually, with affirmative responses scored as 1 and negative responses scored as 0. After the completion of the questionnaire, an overall score (0–14) is calculated by adding up all the individual item scores, and adherence to the MD is classified into three categories (low: ≤5, medium: 6–9, and high: ≥10) of adherence.

The selection of the MEDAS questionnaire to assess MD adherence was due to its widespread use in previous studies and its simple and fast application when compared to semiquantitative food frequency questionnaires, which are commonly used to assess diet. The fact that is has been validated and its effectiveness in assessing adherence to the MD has been proven against other questionnaires with a larger number of items [27] was also considered when choosing the MEDAS questionnaire.

In order to collect accurate information on habitual diet throughout pregnancy, during the last programed antenatal visit (36–38 weeks), women were asked to recall their usual diet during the preceding ~9 months and, with this in mind, instructed to complete the MEDAS questionnaire.

### 2.4. Statistical Analysis

The IBM SPSS Statistics (version 27.0) software was used for data analysis. Categorical variables data are presented as frequencies and percentages and continuous variable data as means and standard deviations. Both are presented with a 95% confidence interval. *p*-values were obtained by χ2 test for categorical variables and by ANOVA for continuous variables, and results were considered statistically significant if the *p*-value < 0.05.

To analyze variable distribution, the Shapiro–Wilk test was performed to identify the normal distribution of the continuous variables. For comparison among two or more groups of continuous variables, the Kruskal–Wallis rank-sum test was performed.

The risk of iron deficiency diagnosis, defined as serum ferritin <30 g/L [20], was assessed using the odds ratio and a 95% confidence interval, taking as the reference level high adherence to the Mediterranean dietary pattern based on the MEDAS score. As an impact measure, the attributable fraction and the 95% confidence interval due to diet for iron deficiency diagnosis was calculated.

## 3. Results

### 3.1. Mediterranean Dietary Pattern Adherence

Of the 506 women who participated in the study, 116 (22.9%) were classified as demonstrating a high adherence (Score ≥ 10), 277 (54.7%) a medium adherence (Score 6–9), and 113 (22.3%) a low adherence (Score 0–5).

### 3.2. Maternal Sociodemographic and Anthropometric Characteristics

As shown in Table 1, the pregnant women had a mean age of between 31 and 35 years, with age being directly associated with MD adherence. Most women were of European origin (82.0%) and the most common educational level achieved was secondary studies (44.1%). Those with higher levels of education presented better adherence to the MD.

Mean height, pre-pregnancy weight, and pre-pregnancy BMI were between 1.62 and 1.64 cm, 63 and 65 kg, and 23 and 24, respectively. Mean weight gained was around 12 kg, and the average BMI in the third trimester was 28.

**Figure 2:**
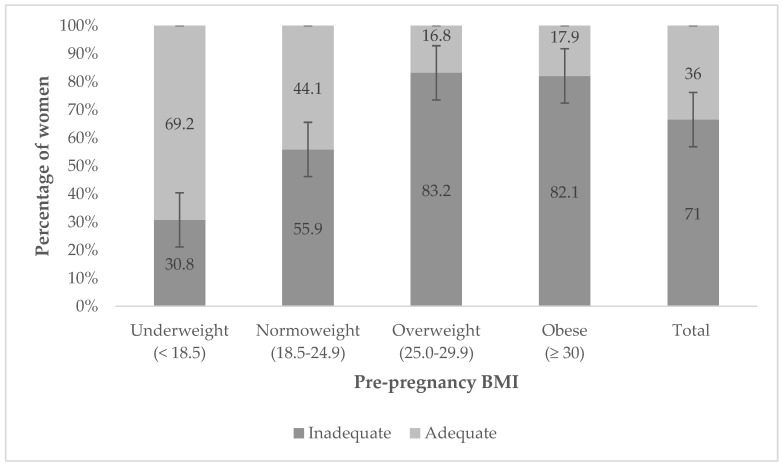
Weight gain adequacy during pregnancy according to pre-pregnancy BMI.

**Figure 3:**
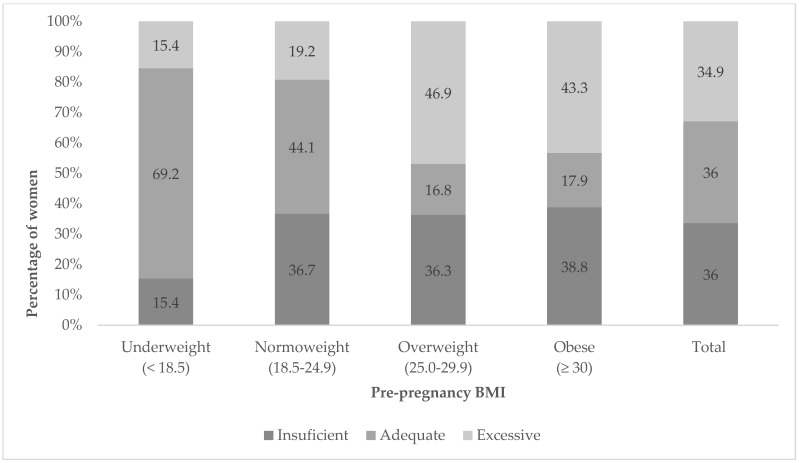
Weight gain classification during pregnancy according to pre-pregnancy BMI.

In all pre-pregnancy BMI category groups, except underweight, more women presented inadequate gestational weight gains (Figure 2). In the overweight and obese categories, over 80% of participants presented inadequate gestational weight gain. If this inadequate weight gain is presented broken down into its two possible categories (insufficient or excessive), it was observed that more of the women in the overweight and obese categories fell into the excessive weight gain group, while the opposite was true for the normoweight category (Figure 3).

**Figure 4:**
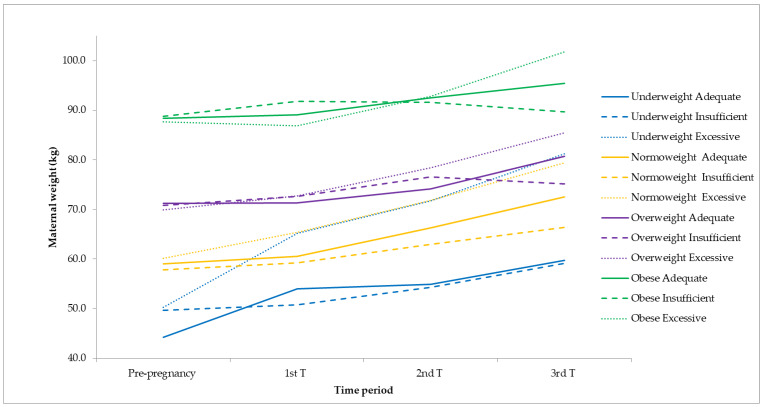
Mean maternal weight according to pre-pregnancy BMI and weight gain adequacy.

Mean maternal weight throughout pregnancy according to pre-pregnancy BMI and weight gain adequacy can be seen in Figure 4.

**Figure 5:**
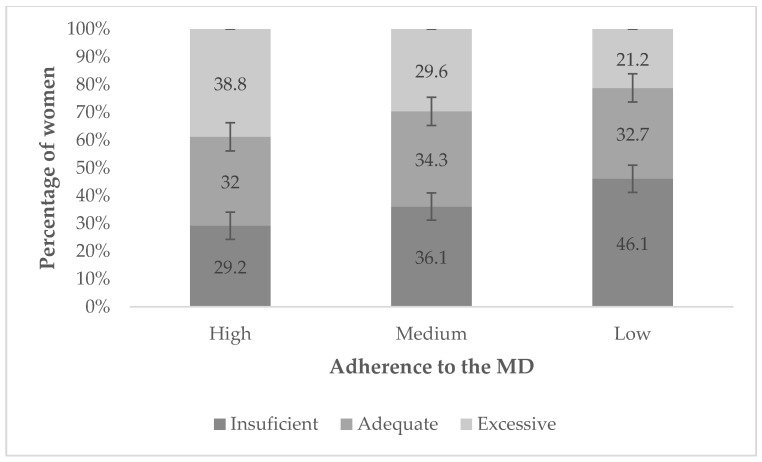
Weight gain adequacy during pregnancy according to adherence to the MD.

While no differences were observed in gestational weight gain among the MD adherence groups, adequacy of weight gain did vary among those with worse adherence to the MD, presenting more cases of insufficient weight gain, while those with high MD adherence presented more cases of excessive weight gain (Figure 5).

The majority of the participating women performed physical activity (both during work and leisure) during pregnancy, although it was mostly of a light intensity. Over 14% of the sample continued to smoke during pregnancy and almost 3% consumed alcohol at some point during pregnancy. Tobacco use was more prevalent in the group with medium adherence to the MD, while alcohol use was associated with the groups with worse MD adherence. When it came to supplement use, a statistically significant difference was observed regarding the use of vitamin (other than folic acid and/or iron) supplements. While no differences were observed in iron supplementation, its use followed an inverse trend of association with MD adherence. No differences were observed in the obstetric history variables collected.

Of all the variables analyzed and identified in Table 1, age, origin, educational level, glucose in the first trimester, gestational weight gain, leisurely physical activity, tobacco use, and vitamin supplementation were all significantly different between groups.

### 3.3. Newborn Characteristics

Table 2 shows the basic characteristics of newborns according to maternal level of adherence to the MD. No significant differences were observed among the groups for any of the studied variables, with the notable exception of mode of delivery. The proportion of caesarean sections was lower in the high adherence group compared to the other groups (28.4% vs. 53.1% and 65.5%), and inversely for vaginal deliveries (71.6% vs. 46.9% and 34.5%).

### 3.4. Iron-Related Biochemical Parameters

The results regarding the iron-related maternal biochemical parameters are presented in Table 3. For hemoglobin and ferritin levels, no differences were observed among the adherence groups during pregnancy. During pregnancy, the only significant difference observed was in red blood cell count, which was higher in women with better adherence to the MD across all three trimesters.

In Table 4, the results of the crude and adjusted odds ratio and attributable fraction calculations are shown. The diagnostic threshold used was of Ferritin level < 30 g/L since there were groups were no women met the Hemoglobin diagnostic threshold, and therefore, calculations were not possible. Given this, the results refer to the risk of iron deficiency and not necessarily anemia. With high adherence to the MD as the reference group, the crude odds of iron deficiency diagnosis were only significant in the first trimester for both the medium [OR = 2.99 (1.55–5.75)] and low [OR = 4.39 (2.15–8.96)] adherence groups, with deficient adherence to the Mediterranean dietary pattern accounting for 66.5% (35.5–82.6) and 77.2% (53.5–88.8) of the risk of iron deficiency diagnosis in the medium and low adherence groups, respectively. The crude results were adjusted for all maternal characteristics and habits and newborn characteristics that presented statistically significant differences among the adherence groups, as shown in Table 1 and Table 2, (maternal age, maternal origin, maternal education level, glucose in first trimester, gestational weight gain, physical activity (leisure), tobacco, vitamin supplement and infant birth weight) as well as an adjustment including only those variables that presented the strongest likelihood of confounding according to their weight in the initial adjusted analysis. After adjustment, significance was lost. This was probably due to both the number of possible confounding factors that were adjusted for and the sample size.

## 4. Discussion

Pregnancy entails an increase in oxygen consumption, and iron plays an essential role in its correct transport and delivery to the maternal–placental–fetal unit. It is, in fact, so fundamental for this process that its mechanism of action is conserved across species. Meanwhile, gestational weight gain is considered a modifiable risk factor for adverse maternal–fetal outcomes and is a global concern, as the proportion of women whose gain falls outside of the recommendations is substantial.

### 4.1. Iron Deficiency

Globally, it is estimated that close to two billion people, or a quarter of the world’s population, are iron deficient, making it the most common micronutrient deficiency in the world [28]. Iron deficiency disproportionally affects women, and in premenopausal women it is mainly due to an imbalance between dietary iron intake and physiologic blood loss incurred during menstruation or previous pregnancy [21,28]. Decreased dietary iron intake can contribute to this imbalance, as can iron malabsorption [29,30].

In low- and middle-income countries, iron deficiency rates can be as high as 80%, whereas in high-income countries rates are estimated to be around 45% depending on the markers and diagnostic thresholds used [28,31]. The NHANES study, which uses a serum ferritin cutoff of 20–30 µg/L, found that at least 25% of 15–39-year-old women were iron deficient [32]. Another study estimated the prevalence of iron deficiency among pregnant women to be anywhere from 16% to 77%, with rates increasing progressively across trimesters [33].

In this study, the prevalence of iron deficiency (ferritin level < 30 g/L) was 23.9%, 72.7%, and 77.7%, respectively, for trimesters one, two, and three. This prevalence of iron deficiency is high and most notably of concern in the second and third trimesters.

Pregnancy increases maternal iron demand for three reasons: maternal plasma and blood volumes are increased; the fetus requires iron for its own metabolic and oxygen delivery needs as well as the loading of its endogenous iron stores; and the placenta is a highly metabolically active organ with large iron requirements [34,35,36].

Iron deficiency during pregnancy is associated with adverse outcomes for both mother and child. During pregnancy, the fetal brain is at particular risk, and low maternal gestational iron has been associated with autism, schizophrenia, and abnormal brain structure in the offspring [34].

Iron deficiency in infants and toddlers is extremely common and until recently was thought to be due to a combination of poor dietary iron intake and blood loss [34]. However, a study demonstrated that iron deficiency was driven to a large degree by neonatal iron status and is therefore a function of fetal iron loading [37].

Previous studies have shown that neonatal iron deficiency negatively affects neurologic and behavioral functions in the neonatal period and confers long-term risks of neurodevelopment [34]. Newborns with iron deficiency have been shown to present slower processing speeds, compromised recognition memory, and poorer bonding that persist despite iron repletion [34].

The results of this study highlight the problem of iron deficiency in the latter stages of pregnancy, as the prevalence during this period was significantly higher than that found during the first trimester and affected around three quarters of the studied women. Complications derived from an untreated iron deficiency can significantly negatively impact the health of both the mother and child.

### 4.2. Anemia

Around 2 to 5% of pregnant women are diagnosed with anemia during the first trimester of pregnancy, while, in developed countries, 10 to 20% of women are diagnosed in the third trimester [23,38], making it a common diagnosis among pregnant women. Even though maternal iron stores are expected to rebound after giving birth, postpartum anemia rates remain high in both developed (22–50%) and developing (50–80%) countries [39].

In this study, anemia (hemoglobin level < 11 g/dL in the first or third trimesters and hemoglobin level < 10.5 g/dL in the second trimester) prevalence was 5.3%, 15.6%, and 12.3%, respectively, for trimesters one, two, and three.

While the prevalence of anemia during pregnancy fell within the expected range, this might still be indicative of a public health problem which may benefit from a public health campaign and/or intervention.

While the prevalence of anemia during the first trimester is low, the risk of diagnosis is three folds higher in women with medium or low adherence to the MD, with over two thirds of the risk attributable to their diets in both cases. These results open the door for a possible nutritional intervention that may optimize maternal iron levels from the early stages of pregnancy and could help improve the rates of anemia in the subsequent trimesters and postpartum.

Anemia is classified as moderate or severe when hemoglobin levels are between 7 and 9 g/dL or less than 7 g/dL, respectively [38]. It can adversely affect maternal–fetal health and has been linked to increased morbidity and mortality [40]. Pregnant women suffering from anemia have been shown to be at an increased risk of perinatal infections, pre-eclampsia, and bleeding, while other symptoms such as breathing difficulties, fainting, tiredness, palpitations, and sleep difficulties are also common [41]. Previous studies have shown that gestational anemia significantly increases the risk of postpartum depression [42], and hemoglobin levels under 9 g/dL during the postpartum period have been shown to be associated with a higher risk of post-traumatic stress disorder [43].

Some of the more common adverse perinatal outcomes associated with maternal anemia include intrauterine growth retardation, prematurity, and low birth weight [44,45,46]. In an infant born to an anemic mother, lowered iron stores can last for up to a year and derive in anemic infants/toddlers [47], which puts them at risk for developmental delays or setbacks. These delays may involve cognitive, social-emotional, and adaptive functions such as language and motor development [48,49]. In normal circumstances, breastfeeding is a protective factor against this, but if the mother is iron deficient, this is not the case to begin with, and it is also known that breast milk iron levels fall over time, so any possible protective effect would be further reduced [50].

It should be noted that the association between maternal hemoglobin levels and adverse outcomes follows a U-shaped curve. Both low (Hb < 11.0 g/dL) and high (Hb > 13.0 g/dL) maternal gestational hemoglobin levels [51,52] are associated with adverse outcomes and any intervention should take this into account [53,54,55]. Although iron is an essential nutrient, in excess it is a pro-oxidant that can derive in deviation from optimal health [56].

Maternal hemoglobin recovery is often delayed [57], but after a normal pregnancy without iron deficiency and a delivery without significant blood loss, absorbed iron requirements decline to ~1.1 mg/day, which should be possible to fulfill through a healthy, balanced diet [39]. In the most favorable conditions, the rate of dietary iron absorption can reach 30% of dietary iron intake, which would mean that an iron intake of 9 mg would correspond to an absorption of 3 mg iron [39]. Therefore, the recommended iron intake while breastfeeding is 8.5–9.0 mg/day for women whose menstruation has not restarted and 10.5 mg/day for those for whom it has [39].

### 4.3. Gestational Weight Gain

Gestational weight gain is an important prognostic factor for short- and long-term health outcomes for the mother and the newborn [58] and represents a potentially modifiable risk factor for adverse outcomes [59]. Both excessive and insufficient gestational weight gain have been associated with adverse maternal and neonatal outcomes, but the risks are different for each type of inadequacy [59]. Many reports have elucidated the risk factors for inadequate gestational weight gain, including pre-pregnancy BMI, race or ethnicity, and socioeconomic status [59].

In this study, seven out of ten women presented inadequate gestational weight gain. Worse adherence to the MD was associated with insufficient weight gain, and optimal adherence with excessive weight gain. Within the pre-pregnancy BMI categories, the underweight category was the only one where at least half of the women met the IOM guidelines for gestational weight gain. It should also be noted that in the overweight and obese categories, merely 20% of women meet these recommendations.

Excessive gestational weight gain is considered to be multifactorial, and even though pre-pregnancy BMI and sedentarism are the main factors, other socio-demographic, economic, psychological, and dietary factors, as well as prenatal care, are also involved [58].

Although insufficient gestational weight gain has also been linked to adverse outcomes [58], its associated factors have been scarcely studied when compared to those associated with excessive gestational weight gain. Evidence suggests that women who begin their pregnancies underweight are unlikely to improve their nutritional status throughout the gestational period and have higher rates of preterm deliveries and stillbirths, as well as underweight and mentally impaired children [60]. In the few studies available, insufficient gestational weight gain has been linked to pre-pregnancy BMI and malnutrition [58]. However, almost no attention has been placed on insufficient gestational weight gain outside of these two factors, probably due to the current obesity epidemic and high prevalence of excessive gestational weight gain, which has attracted the interest of researchers and medical professionals in recent decades.

### 4.4. Interventions

Dietary intervention has the potential to optimize iron levels during pregnancy. While few studies have estimated the specific nutritional value of the MD when it comes to iron, those that do exist consistently report that better adherence to the MD is associated with higher iron intake [61,62,63], and that the components of the MD could also facilitate the elimination of excess iron stores [64]. Iron from food comes in two forms: heme and non-heme. Heme is found only in animal flesh such as meat, poultry, and seafood. Non-heme iron is found in plant foods such as whole grains, nuts, seeds, legumes, and leafy greens, which are all staples of the MD, as are some iron absorption inhibitors such as phytates, polyphenols, and dairy products. In addition to its effect on maternal iron levels, the MD has been shown to be beneficial for the health of both the mother and child, and it is widely considered as an optimal diet to follow during pregnancy [65,66].

Evidence available regarding the prevalence of iron deficiency has led governing bodies to consider whether universal supplementation with iron during pregnancy should be policy [34]. The recommendation that iron-deficient pregnant women should be treated is universally accepted; however, care must be taken with issuing blanket recommendations as there is emerging evidence that suggests an increased risk of glucose intolerance in iron-sufficient pregnant women treated with iron [67].

Multiple vitamin and micronutrient deficiencies often coexist among women of reproductive age, especially during pregnancy, when micronutrient requirements increase [68]. Multiple micronutrient supplements (MMSs)—that is, supplements providing several vitamins and minerals—can fill nutrient gaps for pregnant women and have been shown to be a safe and cost-effective intervention [68]. Most prenatal MMSs, including the United Nations International Multiple Micronutrient Antenatal Preparation (UNIMMAP) [69], contain 30 mg of elemental iron.

However, there is growing evidence of the existence of metabolic interactions between micronutrients, and these interactions may be positive or negative [70]. Significant scientific data on interactions between micronutrients and the coexistence of micro- and macro-nutrient deficiencies is not currently available; therefore, serious considerations should be taken before MMSs are universally recommended to pregnant women [70].

Vitamins B6, B9, and B12 help produce functional red blood cells by participating in the production of hemoglobin. Each hemoglobin molecule contains four heme chemical groups, and vitamins B6, B9, and B12 activate enzymes needed to properly form heme. A deficiency in any of these vitamins prevents healthy red blood cell formation. The size of red blood cells in deficient patients is normal or somewhat smaller, but the hemoglobin content is lower. This means each red blood cell would have less capacity for carrying oxygen, resulting in muscle weakness, fatigue, and shortness of breath.

Low vitamin D status frequently coexists with low iron status, and recent research has shown that vitamin D plays a role in the suppression of hepcidin: the primary regulator of systemic iron homeostasis [71]. Therefore, vitamin D deficiency may be a contributing factor to iron deficiency anemia via direct effects on hepcidin.

Vitamin A deficiency has also been associated with low iron status [72], and joint supplementation has been shown to be more effective than individual treatments with iron or vitamin A alone [73].

Several studies have also considered interventions that may help optimize gestational weight gain, but there is no clear and convincing evidence that such interventions can lead to better health outcomes, and most are not easy to implement in typical settings [59]. 

Further research is warranted regarding determining which interventions could help pregnant women to meet gestational weight gain recommendations, and to understand whether these interventions would improve maternal and neonatal outcomes.

### 4.5. Limitations

This study presents some limitations which should be considered. First, the sample size was not very large; therefore, there is less control for confounding variables. We recommended the sample size be increased for future studies in order to obtain more solid and reliable results. This would also allow for a more accurate calculation of adjusted results, which could present a better picture of the factors most associated with maternal, iron-related biochemical parameters apart from diet. The lack of stability or reliability of the adjusted results in this study highlight the multifactorial nature of diet and iron status parameters. In future studies on the relation between diet and iron-related parameters, careful consideration should be given to the possible confounding factors here identified.

The effect of vitamin supplementation (other than folic acid and/or iron) on iron status should also be further explored due to the growing evidence of the existence of metabolic interactions between micronutrients.

In addition, the collection of data regarding dietary patterns through the use of the MEDAS questionnaire was only performed once, introducing a higher possibility of recall bias.

### 4.6. Conclusions

Adherence to the MD is associated with maternal iron deficiency and/or anemia diagnosis. Iron needs should be considered individually, and dietary intervention and iron supplementation should be prescribed according to need and should be continued until Hb concentrations have stabilized after reaching normal levels. The prevalence of gestational weight gain inadequacy is high and associated with poor adherence to the MD and excess pre-pregnancy weight. Interventions to tackle these modifiable risk factors, which could improve maternal and newborn outcomes, are needed.

## Figures and Tables

**Table 1:** Maternal characteristics and habits according to adherence to the Mediterranean dietary pattern.

	High Adherence (Score ≥ 10) n = 116 (22.9%)	Medium Adherence (Score 6–9) n = 277 (54.7%)	Low Adherence (Score 0–5) n = 113 (22.3%)	
	n (%)/mean ± SD	n (%)/mean ± SD	n (%)/mean ± SD	*p*-Value *
**Age** (years)	34.19 ± 5.38	32.36 ± 5.58	31.42 ± 6.23	0.001
<35 years	65 (56.5%)	192 (69.3%)	77 (68.1%)	0.049
≥35 years	50 (43.5%)	85 (30.7%)	36 (31.9%)	0.049
**Origin**				0.057
European	100 (86.2%)	216 (78.0%)	99 (87.6%)	0.043
American	8 (6.9%)	61 (22.0%)	14 (12.4%)	0.004
Asian	8 (6.9%)	0 (0.0%)	0 (0.0%)	-
**Educational Level**				0.001
No studies	6 (5.2%)	9 (3.2%)	1 (0.9%)	0.178
Primary	12 (10.3%)	34 (12.3%)	21 (18.6%)	0.143
Secondary	37 (31.9%)	134 (48.4%)	52 (46.0%)	0.009
Tertiary	43 (37.1%)	90 (32.5%)	36 (31.9%)	0.629
Post-graduate	18 (15.5%)	10 (3.6%)	3 (2.7%)	0.001
**Height** (m)	1.64 ± 0.06	1.62 ± 0.61	1.62 ±0.58	0.707
**Pre-pregnancy weight** (kg)	64.32 ± 11.85	63.87 ± 13.08	64.92 ± 13.09	0.762
**Weight first trimester** (kg)	63.63 ± 13.00	65.70 ± 13.68	66.92 ± 12.33	0.360
**Weight second trimester** (kg)	68.61 ± 10.98	70.23 ± 13.76	72.00 ± 11.42	0.349
**Weight third trimester** (kg)	75.42 ± 12.44	75.76 ± 13.57	77.89 ± 12.68	0.283
**Gestational weight gain** (kg)	11.14 ± 4.67	11.89 ± 4.85	12.49 ± 4.92	0.122
Insufficient	34 (29.2%)	100 (36.1%)	52 (46.1%)	0.030
Adequate	37 (32.0%)	95 (34.3%)	37 (32.7%)	0.887
Excessive	45 (38.8%)	82 (29.6%)	24 (21.2%)	0.014
**Pre-pregnancy BMI**	23.81 ± 3.74	24.03 ± 5.29	24.48 ± 5.37	0.589
Underweight (≤18.4)	8 (7.0%)	17 (6.2%)	5 (4.5%)	0.713
Normal weight (18.5–24.9)	69 (60.5%)	165 (59.8%)	63 (56.3%)	0.806
Overweight (25.0–29.9)	29 (25.4%)	60 (21.7%)	28 (25.0%)	0.691
Obesity (≥30)	8 (7.0%)	34 (12.3%)	16 (14.3%)	0.137
**BMI first trimester**	27.88 ± 3.87	28.43 ± 5.63	29.45 ± 5.20	0.104
**BMI second trimester**	25.58 ± 3.56	26.84 ± 5.27	27.41 ± 4.33	0.092
**BMI third trimester**	27.69 ± 4.60	28.01 ± 5.63	28.17 ± 3.55	0.961
**Glucose** (mg/dL) **first trimester**	74.22 ± 9.52	77.65 ± 6.26	82.31 ± 10.14	0.028
**Glucose** (mg/dL) **second trimester**	69.71 ± 9.62	74.33 ± 14.65	75.16 ± 8.51	0.611
**Glucose** (mg/dL) **third trimester**	74.50 ± 14.47	79.95 ± 15.68	75.10 ± 6.45	0.172
**O’Sullivan glucose** (mg/dL) **first trimester**	141.33 ± 30.87	119.07 ± 3249	129.28 ± 33.09	0.067
**O´Sullivan glucose** (mg/dL) **second trimester**	145.38 ± 33.80	142.58 ± 48.81	141.67 ± 36.24	0.980
**Physical activity** (leisure)				0.005
None	20 (17.2%)	83 (30.0%)	42 (37.8%)	0.001
Light	76 (65.5%)	162 (58.5%)	60 (53.2%)	0.158
Moderate	20 (17.2%)	27 (9.7%)	8 (7.2%)	0.031
Intense	0 (0.0%)	5 (1.8%)	1 (0.9%)	0.828
**Physical activity** (work)				0.523
None	31 (26.7%)	88 (31.8%)	38 (33.9%)	0.488
Light	85 (73.3%)	187 (67.5%)	74 (66.1%)	0.451
Moderate	0 (0.0%)	2 (0.7%)	0 (0.0%)	-
Intense	0 (0.0%)	0 (0.0%)	0 (0.0%)	-
**Tobacco**	11 (9.6%)	49 (18.2%)	12 (11.0%)	0.046
**Alcohol**	0 (0.0%)	9 (3.3%)	6 (5.3%)	0.160
**Folic acid supplement**	70 (60.3%)	169 (61.2%)	56 (50.0%)	0.114
**Iron supplement**	12 (85.7%)	57 (90.5%)	30 (93.8%)	0.678
**Vitamin supplement**	98 (86.0%)	201 (73.6%)	87 (77.7%)	0.030
**Nutritional supplement**	64 (62.1%)	125 (56.1%)	56 (59.6%)	0.563
**Previous pregnancy loss**	31 (27.0%)	94 (34.2%)	44 (39.3%)	0.065
**Nulliparous**	59 (51.3%)	146 (53.3%)	51 (45.5%)	0.798
**Time between pregnancies** (months)	59.83 ± 6.67	53.24 ± 4.2	56.65 ± 5.8	0.939

The values correspond to the mean and the standard deviation for the quantitative variables, and to the number and frequency for the qualitative variables. * *p*-value obtained by ANOVA (*p* < 0.05) for quantitative variables, and by χ2 test (*p* < 0.05) for qualitative variables.

**Table 2:** Newborn characteristics according to maternal adherence to the Mediterranean dietary pattern.

	High Adherence (Score ≥ 10) n = 116 (22.9%)	Medium Adherence (Score 6–9) n = 277 (54.7%)	Low Adherence (Score 0–5) n = 113 (22.3%)	
	n (%)/mean ± SD	n (%)/mean ± SD	n (%)/mean ± SD	*p*-Value *
**Sex**				0.280
Male	67 (57.8%)	141 (50.9%)	50 (44.2%)	
Female	49 (42.2%)	136 (49.1%)	63 (55.8%)	
**Gestational age** (weeks)	39.24 ± 1.39	39.14 ± 2.04	39.10 ± 1.71	0.821
**Birth weight** (g)	3270.75 ± 400.69	3155.38 ± 487.54	3203.29 ± 593.79	0.247
Low <2500 g	0 (0.0%)	18 (6.5%)	7 (6.2%)	0.911
Average 2500–4000 g	110 (94.8%)	254 (91.7%)	98 (86.7%)	0.089
High >4000 g	6 (5.2%)	5 (1.8%)	8 (7.1%)	0.029
**Length**	50.80 ± 1.47	50.2 1± 1.92	50.22 ± 2.33	0.247
**Head circumference**	34.07 ± 1.31	33.97 ± 1.35	34.09 ± 1.38	0.776
**Newborn classification**				0.659
Small (SGA)	8 (6.9%)	31 (11.2%)	12 (10.6%)	0.425
Adequate (AGA)	95 (81.9%)	223 (80.5%)	86 (76.1%)	0.506
Large (LGA)	13 (11.2%)	23 (8.3%)	15 (13.3%)	0.301
**Vaginal delivery**	83 (71.6%)	130 (46.9%)	39 (34.5%)	0.001
**Cesarean section**	33 (28.4%)	147 (53.1%)	74 (65.5%)	0.001
**Admission to NICU**	2 (1.7%)	2 (0.7%)	0 (0.0%)	0.338
**Admission to SCNU**	11 (9.5%)	16 (5.8%)	7 (6.2%)	0.407

SGA: <10th percentile. AGA: between 10th and 90th percentile. LGA: >90th percentile. NICU: neonatal intensive care unit. SCNU: special care neonatal unit. The values correspond to the mean and the standard deviation for the quantitative variables, and to the number and frequency for the qualitative variables. * *p*-value obtained by ANOVA (*p* < 0.05) for quantitative variables, and by χ2 test (*p* < 0.05) for qualitative variables.

**Table 3:** Iron-related biochemical parameters during the pregnancy according to adherence to the Mediterranean dietary pattern.

	High Adherence (Score ≥ 10) n = 116 (22.9%)	Medium Adherence (Score 6–9) n = 277 (54.7%)	Low Adherence (Score 0–5) n = 113 (22.3%)	
	n (%)/mean ± SD	n (%)/mean ± SD	n (%)/mean ± SD	*p*-Value *
**First trimester**				
Hematocrit %	38.85 ± 2.96	37.73 ± 2.59	37.97 ± 3.22	0.548
Red blood cells 10^12^/L	4.55 ± 0.46	4.21 ± 0.32	4.26 ± 0.36	0.030
Hemoglobin g/dL	12.81 ± 1.00	12.61 ± 095	12.80 ± 1.03	0.727
Hemoglobin level < 11 g/dL	0 (0.0%)	22 (7.9%)	5 (4.4%)	0.594
Iron mcg/dL	96.50 ± 47.39	88.60 ± 34.36	89.00 ± 33.16	0.825
Ferritin g/L	61.70 ± 6.03	55.96 ± 3. 68	41.17 ± 2.20	0.303
Ferritin level < 30 g/L	12 (10.3%)	71 (25.6%)	38 (33.6%)	0.396
**Second trimester**				
Hematocrit %	34.73 ± 2.08	34.29 ± 2.41	34.61 ± 2.42	0.793
Red blood cells 10^12^/L	3.99 ± 0.28	3.72 ± 0.25	3.78 ± 0.30	0.017
Hemoglobin g/dL	11.45 ± 0.90	11.48 ± 0.82	11.59 ± 0.82	0.837
Hemoglobin level < 10.5 g/dL	23 (19.8%)	47 (16.9%)	9 (8.0%)	0.030
Iron mcg/dL	87.67 ± 57.78	87.72 ± 36.6	80.40 ± 2806	0.720
Ferritin g/L	31.42 ± 4.20	25.03 ± 15.70	25.90 ± 18.80	0.703
Ferritin level < 30 g/L	81 (69.8%)	206 (74.3%)	81 (71.7%)	0.994
**Third trimester**				
Hematocrit %	36.91 ± 2.78	36.46 ± 2.25	35.45 ± 3.14	0.131
Red blood cells 10^12^/L	4.22 ± 0.29	3.96 ± 0.26	3.92 ± 0.37	0.007
Hemoglobin g/dL	12.14 ± 1.27	12.18 ± 0.86	11.83 ± 1.19	0.304
Hemoglobin level < 11 g/dL	17 (14.7%)	22 (7.9%)	23 (20.4%)	0.228
Iron mcg/dL	92.64 ± 6.47	90.32 ± 4.13	78.33 ± 4.37	0.549
Ferritin g/L	27.29 ± 22.50	22.31 ± 11.73	22.67 ± 19.09	0.796
Ferritin level < 30 g/L	83 (71.6%)	225 (81.2%)	85 (75.2%)	0.855

The values correspond to the mean and the standard deviation for the quantitative variables, and to the number and frequency for the qualitative variables. * *p*-value obtained by ANOVA (*p* < 0.05) for quantitative variables, and by χ2 test (*p* < 0.05) for qualitative variables.

**Table 4:** Odds ratio and attributable fraction of Ferritin level < 30 g/L in each trimester according to level of adherence to the Mediterranean dietary pattern.

	High Adherence (Score ≥ 10) n = 116 (22.9%)		Medium Adherence (Score 6–9) n = 277 (54.7%)		Low Adherence (Score 0–5) n = 113 (22.3%)	
	Odds Ratio	Attributable Fraction	Odds Ratio	Attributable Fraction (%)	Odds Ratio	Attributable Fraction (%)
**First** **trimester**	Ref	Ref	2.99 (1.55–5.75)	66.6 (35.5–82.6)	4.39 (2.15–8.96)	77.2 (53.5–88.8)
			1.40 (0.09–22.15) *	28.5 (0.0–95.5) *	2.36 (0.12–45.75) *	57.6 (0.0–97.8) *
			2.92 (0.32–26.70) ‡	65.8 (0.0–96.3) ‡	3.86 (0.38–38.98) *	74.1 (0.0–97.4) ‡
**Second trimester**	Ref	Ref	1.25 (0.78–2.03)	20.2 (0.0–50.6)	1.09 (0.62–1.93)	8.6 (0.0–48.3)
			4.13 (0.40–43.07) *	75.8 (0.0–97.7) *	3.09 (0.20–47.40) *	67.6 (0.0–97.9) *
			1.23 (0.26–5.70) ‡	18.7 (0.0–82.5) ‡	1.06 (0.20–5.64) ‡	5.7 (0.0–82.3) ‡
**Third** **trimester**	Ref	Ref	1.72 (1.04–2.85)	41.9 (3.8–64.9)	1.21 (0.67–2.17)	17.1 (0.0–54.0)
			0.03 (0.00–3002.03) *	0.0 (0.0–100.0) *	19,687.84 (0.32–1,205,893,473.80) *	100.0 (0.0–100.0) *
			2.68 (0.28–25.87) ‡	62.7 (0.0–96.1) ‡	1.83 (0.19–17.66) ‡	45.4 (0.0–94.3) ‡

* Adjusted for maternal age, maternal origin, maternal education level, glucose in first trimester, physical activity (leisure), tobacco, vitamin supplement, gestational weight gain, and infant birth weight. ‡ Adjusted for maternal age, physical activity (leisure), tobacco, vitamin supplement, gestational weight gain, and infant birth weight.

## Data Availability

Not applicable.
